# Transcription Factors GATA1/2 in Hematological Disorders

**DOI:** 10.1002/jha2.70258

**Published:** 2026-04-24

**Authors:** Matthew Karr, Neil Palmisiano, Xiaoyi Chen

**Affiliations:** ^1^ Department of Hematology and Oncology Robert Wood Johnson Medical School Rutgers University New Brunswick New Jersey USA; ^2^ Division of Blood Disorders Rutgers Cancer Institute of New Jersey New Brunswick New Jersey USA

**Keywords:** acute myeloid leukemia, cytopenias, GATA, myelodysplastic syndrome, transcription factors

## Abstract

**Background:**

GATA1 and GATA2 are zinc‐finger transcription factors essential for normal hematopoiesis. As genetic testing becomes more widely integrated into clinical practice, *GATA1/2*‐related disorders are increasingly recognized, making it important for clinicians to understand their diagnosis and management.

**Aims:**

This review summarizes the clinical features, disease mechanisms, and management considerations for *GATA1*‐ and *GATA2*‐related hematological disorders.

**Content:**

We discuss germline *GATA1* mutations causing rare X‐linked erythroid and megakaryocytic cytopenias, somatic *GATA1* mutations driving myeloid leukemia of Down syndrome, and germline *GATA2* mutations causing *GATA2* deficiency syndrome—a predisposition to immunodeficiency and myeloid malignancies affecting up to 75%–80% of carriers. Evolving genotype–phenotype patterns, the somatic mutational landscape, and current therapeutic strategies, including allogeneic hematopoietic stem cell transplantation (HSCT), are reviewed.

**Summary:**

Despite growing recognition of *GATA1/2*‐related disorders, many aspects of disease biology and clinical variability remain incompletely understood. Earlier identification and risk stratification of affected patients, along with advances in transplant approaches and novel therapeutics, will be essential for improving outcomes.

**Trial Registration:**

The authors have confirmed clinical trial registration is not needed for this submission

## 1 Introduction

The GATA family consists of transcription factors characterized by conserved zinc finger DNA‐binding domains that specifically recognize GATA elements (A/TGATAA/G) in DNA sequences [1]. There are six known GATA transcription factors in vertebrates, subdivided into two groups based on tissue expression profiles: *GATA1/2/3* and *GATA4/5/6*. GATA1/2/3 are primarily distributed in mesoderm‐ and ectoderm‐derived tissues (e.g., hematopoietic system and central nervous system), while GATA4/5/6 are mainly involved in endoderm‐ and mesoderm‐derived tissues (including cardiovascular development and epithelial cell differentiation) [1]. This review focuses on GATA1 and GATA2 because these two factors have the most critical and well‐characterized roles in hematopoiesis and are the primary GATA family members implicated in human blood disorders [2].

*GATA1*, located on the long arm of the X chromosome, regulates a wide range of genes across multiple hematopoietic lineages, including erythrocytes, megakaryocytes, mast cells, basophils, and eosinophils [3]. GATA1 is essential for red blood cell (RBC) development: *GATA1*‐null mice die in the embryonic stage due to anemia, with erythroid cells arrested at the proerythroblast stage and undergoing apoptosis [4]. Subsequent studies have shown that GATA1 promotes erythroid differentiation through the “GATA switch,” wherein GATA1 represses *GATA2* to permit normal erythroid maturation [5]. In megakaryocytes, conditional loss of GATA1 leads to an accumulation of immature megakaryocytes with resultant severe thrombocytopenia [6]. In humans, inherited missense mutations of *GATA1* cause several rare X‐linked disorders characterized by cytopenias, such as X‐linked thrombocytopenia (XLT), X‐linked thrombocytopenia with thalassemia (XLTT), and congenital erythropoietic porphyria (CEP) [7]. Somatic mutations in *GATA1* are associated with transient myeloproliferative disorder (TMD) and acute megakaryoblastic leukemia (AMKL) in individuals with Down syndrome [8].

GATA2 is another critical GATA family member for normal hematopoiesis. *GATA2* knockout in mouse models results in embryonic lethality due to failure of definitive hematopoiesis [9]. Germline heterozygous *GATA2* mutations lead to *GATA2* deficiency syndrome, which presents with a wide range of symptoms, including immunodeficiency with recurrent infections, myeloid malignancies, lung disease, and lymphedema [10]. Over 150 pathogenic germline variants of *GATA2* have been reported; however, correlations between specific variants and clinical phenotypes remain unclear, with the exception of lymphedema (which requires *GATA2* haploinsufficiency) [11]. Overall, individuals with *GATA2* deficiency face a very high lifetime risk of developing myeloid malignancies at a young age, with 75% of carriers affected by a median age of 17 years [12]. In pediatric populations, germline *GATA2* mutations are the most common predisposing factor for myelodysplastic syndrome (MDS) [12]. Common additional cytogenetic abnormalities and somatic mutations associated with GATA2‐related myeloid malignancies include monosomy 7, der(1;7), trisomy 8, as well as mutations in *ASXL1*, *SETBP1*, *STAG2*, and *RUNX1* [13]. These genomic alterations likely contribute to leukemogenesis, though the exact mechanisms remain to be elucidated [13]. Treatment for symptomatic *GATA2* deficiency is individualized, with bone marrow transplantation being the only curative option currently [14] (Table 1).

**TABLE 1:** Germline versus somatic *GATA1* and germline *GATA2* alterations associated with hematologic disease.

Entity	Genetic context	Predominant phenotype	Canonical disease entities	Key clinical implications
*GATA1* (germline)	X‐linked; region‐dependent effects: exon 2 loss of full‐length GATA1 favors erythroid‐predominant severe congenital anemia, while exon 4/N‐ZF variants disrupt FOG1 interaction and favor thrombocytopenia	Erythroid and megakaryocytic cytopenias	XLT, XLTT, CEP; “DBA‐like” severe congenital hyporegenerative anemia with reticulocytopenia (GATA1s‐only variants)	Supportive care; HSCT can be curative in severe cases (case reports). Genetic counseling; prenatal/preimplantation testing encouraged for heterozygous females
*GATA1* (somatic, *GATA1s*)	Somatic exon 2 splice/start‐loss variants producing GATA1s; in trisomy 21, often after TMD	DS‐context transient myeloproliferation and megakaryoblastic leukemia biology	TMD and ML‐DS; ML‐DS has cooperating mutations and marked cytarabine sensitivity (reduced cytidine deaminase)	Generally favorable prognosis with intensive chemotherapy (5‐year EFS > 80%); relapsed/refractory disease remains difficult, especially after HSCT failure
*GATA2* (germline, *GATA2* deficiency syndrome)	Germline heterozygous; incomplete penetrance/variable expressivity (≈50% symptomatic by 20; up to 95% by 60)	Bone marrow failure plus immunodeficiency (B‐cell loss; NK/DC defects; atypical mycobacterial/fungal infections)	MonoMAC, DCML deficiency, Emberger, WILD, chronic neutropenia, familial MDS/AML, primary childhood MDS	High risk of MDS/AML (up to 75%–80%, median onset ∼17 year) with characteristic lesions (monosomy 7, der(1;7), trisomy 8) and acquired mutations (*STAG2*, *ASXL1*, *SETBP1*, *RUNX1*). HSCT is the only curative therapy; surveillance and donor/relative germline testing emphasized

Abbreviations: AMKL, acute megakaryoblastic leukemia; CEP, congenital erythropoietic porphyria; DCML, dendritic cell, monocyte, B and NK lymphoid deficiency; DS, Down syndrome; EFS, event‐free survival; HSCT, hematopoietic stem cell transplantation; MDS, myelodysplastic syndrome; ML‐DS, myeloid leukemia of Down syndrome; NK, natural killer; TMD, transient abnormal myelopoiesis; XLT, X‐linked thrombocytopenia; XLTT, X‐linked thrombocytopenia with thalassemia.

## 2 Normal Function of GATA1

GATA1 is a master transcription factor that orchestrates erythropoiesis and megakaryopoiesis. Germline knockout of *GATA1* in mice results in embryonic lethality between Days 10.5 and 11.5 due to profound anemia, reflecting a block at the proerythroblast stage and subsequent apoptosis of erythroid precursors [15]. In human erythropoiesis, *GATA1* expression is dynamically regulated: it is low in early progenitors, peaks at the proerythroblast stage, and declines during terminal maturation—a pattern essential for normal erythroid differentiation [15].

In addition to erythropoiesis, lineage‐specific knockout models have demonstrated that GATA1 is required for megakaryocyte, eosinophil, and mast cell development [16]. Structurally, GATA1 contains two zinc‐finger DNA‐binding domains. The C‐terminal zinc finger mediates DNA binding, while the N‐terminal zinc finger interacts with key cofactors such as FOG1 to modulate target gene specificity [17]. Both domains are required for normal transcriptional activation; deletions in either region impair downstream gene expression despite intact DNA‐binding ability [18]. As a result, mutations in different regions of *GATA1* produce distinct clinical phenotypes, described below.

## 3 *GATA1* Mutations and Hematological Disorders

*GATA1* mutations give rise to a broad spectrum of inherited and acquired hematologic disorders. Germline mutations typically affect erythroid and megakaryocyte development, whereas somatic *GATA1s* mutations (exon 2 splice or start‐loss variants) drive transient abnormal myelopoiesis (also called transient leukemia of Down syndrome, TMD) and myeloid leukemia of Down syndrome (ML‐DS) [19]. Reduced *GATA1* expression has also been implicated in marrow fibrosis and myeloproliferative disease [20].

*GATA1* genotype strongly influences phenotype. Exon 2 mutations that abolish or reduce full‐length GATA1 favor an erythroid‐predominant disease (severe congenital anemia), while exon 4 mutations impair N‐ZF/FOG1 interactions and predominantly cause thrombocytopenia with variable anemia [21].

Historically, several exon 2 *GATA1* mutations were described as “Diamond–Blackfan anemia (DBA)‐like.” Recent consensus guidelines now recognize *GATA1* as a DBA gene in cases where specific variants produce a classic DBA clinical phenotype—namely, severe congenital hyporegenerative anemia with reticulocytopenia [22]. This classification applies particularly to start‐loss and splice variants that eliminate full‐length GATA1 and produce exclusive GATA1s expression. Notably, unlike ribosomopathies in which DBA is usually caused by ribosomal protein mutations, *GATA1*‐associated DBA results from impaired transcription factor dosage rather than ribosomal dysfunction. Thus, while the clinical phenotype may overlap with classic DBA, the underlying biology is distinct [23, 24]. Accordingly, throughout this review, we use the term “DBA phenotype” rather than classifying all *GATA1*‐related anemia as classical DBA.

Multiple reports describe start‐loss mutations—notably c.2T>C and c.3G>A—that abolish the initiating methionine and lead to exclusive GATA1s expression. These variants cause severe hyporegenerative anemia from infancy (often transfusion‐dependent), closely mimicking DBA [25]. Reported cases include five unrelated patients across different studies [25]. Other exon 2 splice‐altering variants (e.g., c.220G>C) similarly produce exclusive GATA1s and result in transfusion‐dependent anemia in affected males [24, 26, 27, 28]

Missense mutations within the N‐terminal zinc finger—such as V205M, G208R, and D218Y—impair FOG1 interaction and primarily manifest as XLT or XLTT [29]. These disorders feature platelet dysfunction, variable anemia, and dysmegakaryopoiesis.

Somatic exon 2 mutations that produce the short isoform (GATA1s) are essential initiating events in ML‐DS. Although ML‐DS and non‐DS AMKL share megakaryoblastic morphology, they are biologically distinct leukemias. ML‐DS occurs only in the context of trisomy 21 (Down syndrome), often following TMD, and features unique cooperating mutations (e.g., in chromatin modifiers) and marked cytarabine sensitivity due to reduced cytidine deaminase expression [30]. With contemporary risk‐adapted therapy, ML‐DS achieves ∼80% 5‐year event‐free survival—far superior to outcomes in fusion‐driven non‐DS AMKL [30]. Relapsed or refractory ML‐DS, however, remains difficult to treat, particularly after HSCT failure [31].

Recent studies describe germline *GATA1s* mutations that, after acquisition of somatic trisomy 21, produce leukemias clinically indistinguishable from ML‐DS [32]. One report documented a male infant with a germline *GATA1* mutation who acquired trisomy 21 and monosomy 7 and subsequently developed MDS [32]. These cases broaden recognition of *GATA1* as a predisposition gene for Down syndrome–like myeloid neoplasms even in persons without constitutional trisomy 21 [33, 34].

Although many heterozygous females exhibit mild or no symptoms due to balanced X‐inactivation, rare cases demonstrate severe disease. For example, skewed X‐inactivation in combination with a somatic event has led to AMKL‐like disease in female carriers, notably with acquired trisomy 8 as a cooperating lesion [35]. These observations extend the recognized phenotype spectrum and highlight the need for careful family screening when *GATA1* mutations are present [33, 34].

Reduced GATA1 protein expression—often with preserved mRNA levels—has been observed in primary myelofibrosis (PMF) [36]. In murine models, decreased *GATA1* expression induces megakaryocytic hyperplasia and progressive marrow fibrosis, recapitulating features of myeloproliferative neoplasms [36]. Although the mechanistic link remains incompletely defined, these findings suggest that GATA1 insufficiency contributes to fibrotic marrow remodeling.

## 4 Normal Function of GATA2

GATA2 is highly expressed in hematopoietic stem cells (HSCs) and is essential for their generation, maintenance, and expansion. In the absence of the *GATA2* gene, mouse embryos fail to survive beyond Day 11.5, dying with empty yolk sac blood vessels indicative of absent definitive hematopoiesis [37]. Mice with heterozygous deletion of *GATA2* are viable but exhibit compromised HSC function, as demonstrated by poor competitive repopulation and increased apoptosis of *GATA2* ^+/−^ cells [38]. Unlike GATA1—which is critical for erythroid differentiation—GATA2 is dispensable for terminal differentiation of erythroid and granulocyte lineages, but is required for mast cell development [39]. In humans, germline heterozygous mutations of *GATA2* lead to *GATA2* deficiency syndrome, manifesting as cytopenias due to loss of multilineage progenitors along with predisposition to myeloid malignancies [40]. Interestingly, *GATA2* overexpression has been observed in sporadic MDS, suggesting that precise regulation of *GATA2* is crucial [40].

Beyond its role in hematopoiesis, GATA2 is also necessary for lymphovascular development. Conditional knockout of *GATA2* in the endothelial system of mouse embryos leads to defective lymphatic development and edematous embryos [41]. Furthermore, heterozygous *GATA2* mutations in mice result not only in abnormal HSC activity but also in a disrupted endothelial transcriptome and loss of vascular integrity [41]. These findings highlight GATA2's role in maintaining the hematopoietic niche and vascular integrity.

## 5 *GATA2* Mutations in Hematological Malignancies

Germline *GATA2* mutations cause *GATA2* deficiency syndrome, a highly penetrant predisposition to bone marrow failure, immunodeficiency, and myeloid malignancies. More than 500 affected individuals have been described, with over 150 distinct pathogenic variants identified across > 300 families [42]. The clinical spectrum includes MonoMAC syndrome, DCML deficiency, Emberger syndrome, WILD syndrome, chronic neutropenia, familial MDS/AML, and primary childhood MDS [43]. These heterogeneous presentations reflect both incomplete penetrance and variable expressivity: approximately 50% of mutation carriers develop symptoms by age 20, and up to 95% by age 60 [44, 45].

Pathogenic *GATA2* variants include frameshift, nonsense, splice‐disrupting, regulatory‐region, and missense mutations, as well as pathogenic synonymous variants that can impair mRNA splicing or stability [15, 46]. Although broad genotype–phenotype rules remain limited, recent multi‐cohort analyses demonstrate emerging patterns. In the largest combined cohort to date, null (truncating) variants were associated with earlier onset of MDS/AML and higher risk of clonal evolution compared with non‐null alleles [47]. The same study outlined the somatic genomic landscape of *GATA2*‐deficient MDS/AML, with frequent acquisition of mutations in *STAG2*, *ASXL1*, *SETBP1*, and *RUNX1*, supporting a multistep leukemogenic model [47].

*GATA2*‐related MDS displays characteristic cytogenetic abnormalities that differ by age. While monosomy 7, trisomy 8, and der(1;7) are among the most frequent lesions overall, a key distinction is that monosomy 7 predominates in pediatric disease, whereas trisomy 8 is more common in adults [48]. These patterns contrast with sporadic MDS, where del(5q) is common but rarely observed in *GATA2* deficiency [49, 50].

The most consistent immunologic abnormality in *GATA2* deficiency is profound loss of B cells, including naïve and memory subsets, and depletion of B‐cell precursors in blood and marrow [51]. Although monocytopenia is frequently observed, it is not universal; some patients demonstrate normal monocyte counts or even monocytosis [52]. NK‐cell and dendritic‐cell deficiencies, impaired antiviral immunity, and susceptibility to mycobacterial and fungal infections are also typical.

Myeloid malignancies—including MDS, AML, and occasionally CMML—occur in up to 75%–80% of *GATA2* mutation carriers, with a median age of onset around 17 years [53]. In a large pediatric MDS cohort (Wlodarski et al., 2016), *GATA2* was the most common germline predisposition, accounting for 15% of advanced MDS and 7% of all primary childhood MDS cases [53]. *GATA2*‐related MDS often presents with hypocellular marrow, megakaryocytic dysplasia, and increased fibrosis [53]. AML typically arises as a progression from a preceding MDS, although de novo cases can occur [54].

Although uncommon, *GATA2* haploinsufficiency can be associated with myelofibrosis. For example, a germline *GATA2*‐N317S mutation was reported in a patient who developed JAK2‐V617F‐positive PMF, suggesting that GATA2 dysfunction may contribute to a broader myeloid phenotype spectrum [55, 56].

## 6 Treatment and Outcomes in *GATA1* Mutated Hematopoietic Disorders

Inherited *GATA1* mutations are rare and managed based on the severity of clinical symptoms. Supportive treatment is often used in non‐severe cases, including platelet transfusion for clinically significant bleeding, RBC transfusion for elective surgery or symptomatic anemia, iron chelation in recipients of recurrent RBC transfusions, and antibiotic prophylaxis in severely neutropenic patients [52]. In more severe cases, hematopoietic stem cell transplantation (HSCT) can be curative, with good outcomes reported in young recipients in case reports [57]. Genetic counseling is recommended for family members of patients with *GATA1* mutations. Inheritance is X‐linked, meaning that males with pathogenic variants will be affected, while heterozygous females may experience mild to moderate symptoms. For families with a *GATA1* mutation, prenatal or preimplantation genetic testing in heterozygous females of childbearing age is encouraged [27, 58].

In cases of ML‐DS driven by somatic *GATA1* mutations, the prognosis is generally favorable: 5‐year event‐free survival exceeds 80% with intensive chemotherapy [59]. However, outcomes for relapsed or refractory ML‐DS remain dismal—particularly after HSCT failure—highlighting the need for novel therapies and clinical trials to improve survival [60, 61, 62].

## 7 Treatment and Outcomes of *GATA2* Deficiency

Allogeneic HSCT is the only curative therapy for *GATA2* deficiency. Transplantation can restore immune function, reverse organ damage, and eradicate clonal myeloid disease. An expanding body of literature—including several major multicenter HSCT cohorts—now guides clinical decision‐making in this condition [63, 64].

Two of the largest studies to date evaluated outcomes in children and adolescents with germline *GATA2* mutations undergoing HSCT, including a large EWOG‐MDS analysis and other multicenter transplant cohorts [64, 65]. Collectively, these studies demonstrated that HSCT yields long‐term survival rates exceeding 70%–80%, with improved outcomes when performed before the onset of advanced MDS/AML, severe infections, or irreversible organ dysfunction. Reduced‐intensity and non‐myeloablative conditioning approaches were feasible in selected patients, and donor source influenced outcomes, with the best results typically seen with HLA‐identical donors. These findings complement more recent analyses reporting 4‐year OS and EFS of approximately 85% and 82%, respectively [63].

The optimal timing of HSCT remains debated, but most experts now favor a proactive approach given the high lifetime incidence of myeloid malignancies in *GATA2* deficiency. While watchful surveillance is reasonable for patients with stable blood counts and no high‐risk cytogenetic abnormalities, the lifetime risk of abrupt progression remains substantial. Therefore, initiating a donor search at the time of diagnosis should be considered standard practice—especially in pediatric and young adult patients—rather than waiting for overt progression [66, 67].

For individuals not undergoing immediate HSCT, recommended surveillance includes periodic assessment of peripheral blood counts, annual bone marrow examinations with cytogenetics and molecular testing, evaluation of immune function, and screening for HPV‐related malignancies. Supportive care focuses on infection prevention and management of cytopenias [66, 67].

Genetic testing of first‐degree relatives is essential, particularly when considering related donors. Several reports have documented donor‐derived MDS/AML arising from asymptomatic *GATA2* mutation carriers who were unknowingly used as allogeneic donors [68]. Because penetrance is incomplete and expressivity variable, familial clustering may not be clinically evident. Thus, germline testing is recommended for all pediatric, adolescent, and young adult patients with MDS/AML to identify occult predisposition syndromes [69, 70, 71].

## 8 Conclusion and Future Directions

GATA1 and GATA2 are both essential transcription factors in normal hematopoiesis. Both germline and somatic mutations of *GATA1* or *GATA2* cause hematological disorders. Inherited *GATA1* mutations are very rare, whereas heterozygous *GATA2* mutations are among the most common germline mutations in pediatric MDS/AML. While more *GATA1* and *GATA2* mutations are being discovered, the underlying disease mechanisms—such as genotype–phenotype correlations in *GATA2* deficiency—remain challenging to fully elucidate. Optimizing disease management in *GATA2* deficiency, including timing of HSCT, establishing clinical surveillance guidelines, and exploring emerging therapies (such as gene editing), represents an important future direction of research.

## Author Contribution

Matthew Karr and Xiaoyi Chen jointly wrote the manuscript. Matthew Karr, Xiaoyi Chen, and Neil Palmisiano reviewed and edited the draft.

## Funding

The authors have nothing to report.

## Ethics Statement

The authors have nothing to report.

## Consent

The authors have nothing to report.

## Conflicts of Interest

The authors declare no conflicts of interest.

## Data Availability

The authors have nothing to report.
